# Misdiagnosed Histiocytic Sarcoma: A Case Report

**DOI:** 10.1002/ccr3.71104

**Published:** 2025-10-04

**Authors:** Ling Zhang, Jing Qin, Peiwu Li

**Affiliations:** ^1^ Emergency Center Lanzhou University Second Hospital Lanzhou China; ^2^ Emergency Medical Key Laboratory Lanzhou University Second Hospital Lanzhou China; ^3^ Gansu Province Emergency Medicine Clinical Research Center Lanzhou China

**Keywords:** histiocytic sarcoma, inflammation, misdiagnosis, pathological diagnosis

## Abstract

We report a 31‐year‐old male patient repeatedly hospitalized for mimicking infectious symptoms. Following standardized anti‐infective and anti‐inflammatory treatment, clinical evolution was discordant, characterized by treatment non‐response and symptom exacerbation. Ultimately, the multidisciplinary team and repeated histopathological examinations diagnosed histiocytic sarcoma (HS). Unfortunately, the treatment opportunity was lost due to HS's high aggressiveness and rapid progression.

**Summary**

- Histiocytic sarcoma presents with diverse clinical manifestations that often mimic infections or autoimmune diseases, leading to frequent misdiagnoses.
- Molecular pathology is critical for definitive diagnosis and should be pursued when standard treatments fail.

## 1 Introduction

Histiocytic sarcoma (HS) is a rare solid malignancy of hematopoietic origin [1], arising from histiocytes and non‐Langerhans dendritic cells, representing < 1% of hematopoietic malignancies [2]. HS demonstrates a male predominance [3, 4] and wide age distribution, typically presenting as painless extranodal masses (skin, soft tissue, lungs, GI tract, spleen, or liver) with possible nodal involvement [5]. Patients frequently present with systemic symptoms (such as fever, weight loss, fatigue) and lymphadenopathy. HS is highly aggressive, with 70% of cases presenting at advanced stages (III/IV) and demonstrating poor survival (median < 2 years) [6]. The diagnosis primarily relies on histopathological examination.

We report a diagnostically challenging HS case featuring misleading clinical presentations, prolonged hospitalization, treatment refractoriness, and a fatal outcome. Through this case and literature review, we highlight HS's diagnostic challenges and clinical heterogeneity to improve recognition of this rare malignancy.

## 2 Case History and Examination

**FIGURE 1:**
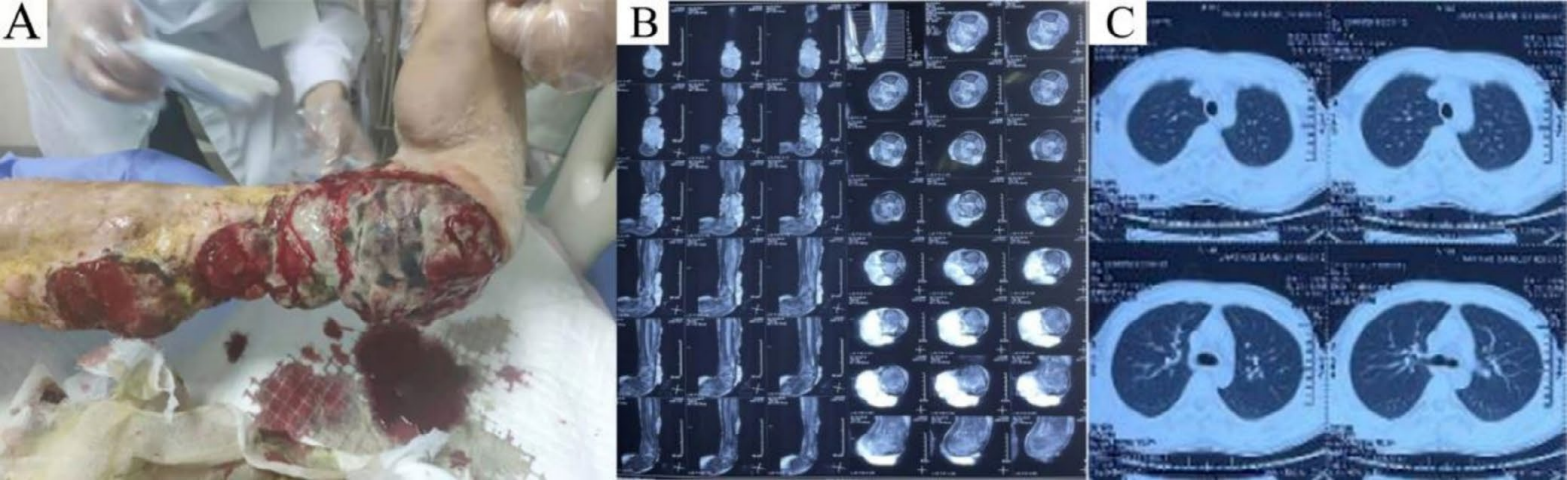
Pre‐amputation clinical and imaging features of the left lower limb. (A) Preoperative skin lesions on the left lower limb. (B) Preoperative MRI of the left lower limb. (C) Preoperative non‐contrast CT scan of the lungs.

At the time of initial presentation, a 31‐year‐old male with no adverse habits and chronic medical history (smoking/alcohol use negative; no hypertension, diabetes, asthma, allergic rhinitis, etc.) presented to the emergency department with cough, expectoration, and high fever (*T*_max_ 39°C). These symptoms appeared 1 month after the amputation of “osteomyelitis of the left lower limb” (Figure 1A–C). Serial peripheral blood analyses (pre‐ and post‐amputation) are detailed in Table 1. Notably, at 15 years of age, the patient developed a small left calf nodule that gradually ruptured, forming an abscess, and he underwent multiple debridements at a local hospital. At 31 years old, his left lower limb skin ulceration worsened with massive exudate; the local hospital diagnosed left lower limb osteomyelitis, prompting left lower limb amputation.

**TABLE 1:** Positive laboratory test results.

		WBC (~10^9^/L)	NE (%)	EO (~10^9^/L)	EO (%)	CRP (mg/L)	IL‐6 (pg/mL)	ESR (mm/h)
Before amputation		42.1	0.8	1.03		120.4		
After amputation		29.1	0.8	1.93		96.4	17.8	
During emergency department admission	Day 1	32.57	0.8	2.23		92.97	23.45	49
	Days 2	50.37	0.87	2.84	0.6	137.1		
	Days 10	31.89	0.81	2.25	0.8	122.37		
	Days 16	32.58	0.81	2.13	0.7	102		
	Days 24	40.1	0.82	3.04	0.8			
	Days 32	40.7	0.85	0.08	0	8.92		
1st follow‐up	Days 61	66.25	0.92	2.77				
2nd follow‐up	Days 80	81.45	0.93	0.12				

**FIGURE 2:**
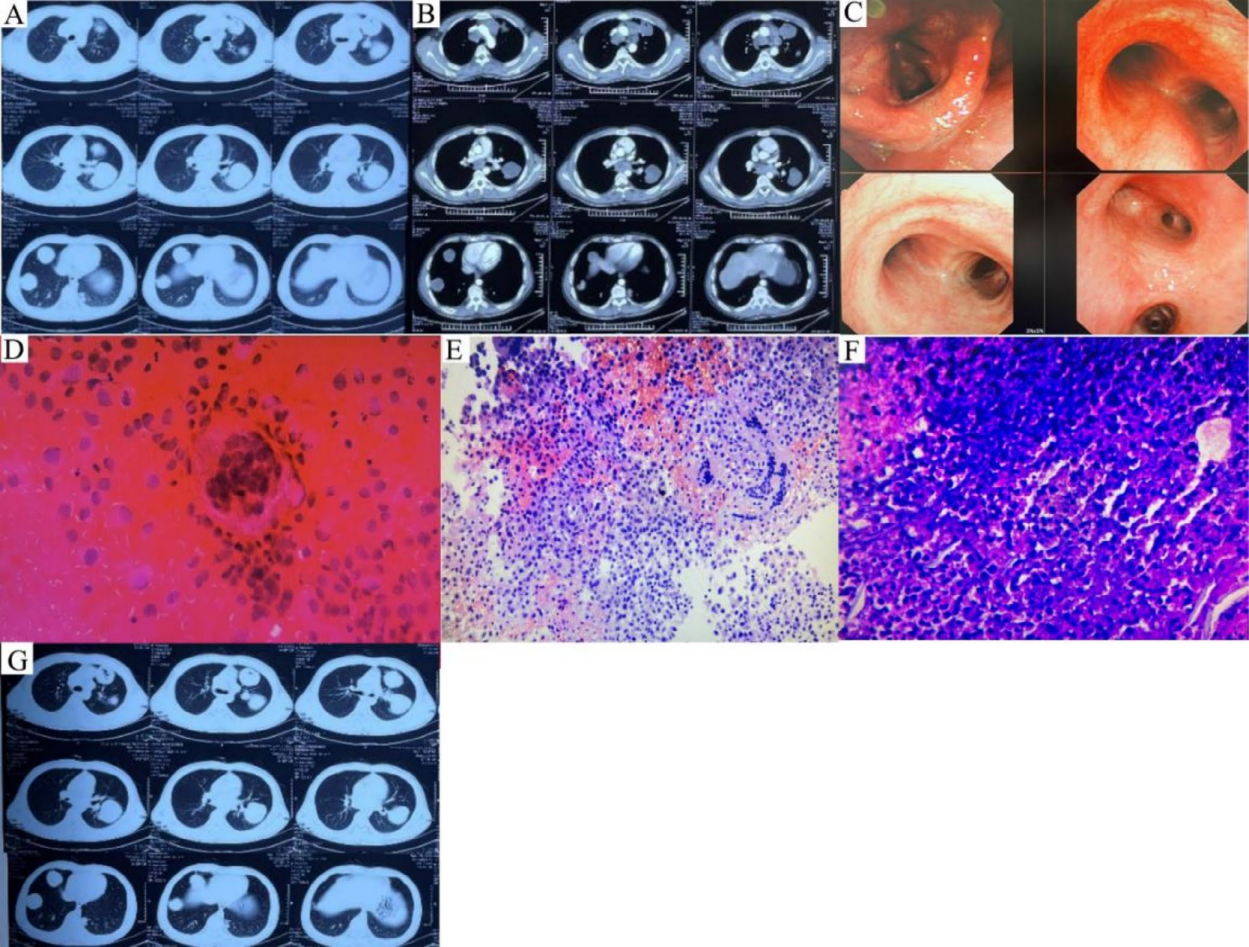
Auxiliary examinations during the hospitalization in the emergency department. (A) Non‐contrast thoracic CT. (B) Contrast‐enhanced thoracic CT. (C) Bronchoscopic findings. (D) Cytological examination of bronchial brush specimens (Hematoxylin and Eosin staining, ×400). (E) Histopathological section of the mediastinal mass (zone 7, Hematoxylin and Eosin staining, ×200). (F) Pulmonary tissue histology (Hematoxylin and Eosin staining, ×200). (G) Follow‐up non‐contrast thoracic CT (Days 80).

On admission (Day 1), initial workup including respiratory examination, sputum culture, and extensive laboratory investigations (comprehensive metabolic panel, coagulation studies, tumor markers, fungal biomarkers [G+GM], blood cultures, tuberculosis diagnostics [Xpert MTB/RIF, T‐SPOT.TB], autoantibody panel, and echinococcus serology) was unremarkable. However, complete blood count showed marked leukocytosis (32.57 × 10^9^/L) with left shift (80% neutrophils) and elevated monocytes (MO: 1.7 × 10^9^/L), eosinophils (EO: 2.23 × 10^9^/L), and basophils (BA: 0.15 × 10^9^/L). Inflammatory markers were significantly elevated: C‐reactive protein (CRP) 92.97 mg/L, interleukin‐6 (IL‐6) 23.45 pg/mL, and erythrocyte sedimentation rate (ESR) 49 mm/h. Thoracic CT (Figure 2A) revealed multiple parenchymal lesions with mediastinal lymphadenopathy, concerning for metastatic involvement. The emergency team's working diagnosis was multiple lung abscesses from Gram‐negative or Gram‐positive pathogens, prompting immediate initiation of broad‐spectrum antimicrobials (Vancomycin 1 g Q12 h, Piperacillin Sodium Tazobactam 4.5 g Q8 h).

Days 2–15: serial complete blood counts demonstrated progressive leukocytosis (WBC: 50.37 × 10^9^/L) with neutrophilia (NE%: 0.87), eosinophilia (EO: 2.84 × 10^9^/L; EO%: 0.6), and elevated CRP (137.1 mg/L). Contrast‐enhanced chest CT (Figure 2B) revealed multiple cystic pulmonary lesions without significant enhancement, accompanied by mediastinal lymphadenopathy. Differential diagnoses included multiple lung abscesses, a mucinous metastatic tumor, and pulmonary hydatid disease. Bronchoscopic examination revealed inflammatory changes in the bronchial mucosa (Figure 2C). Metagenomic next‐generation sequencing (NGS) of bronchoalveolar lavage fluid identified *Streptococcus sanguinis* (sequence number: 402) and cytomegalovirus (CMV; sequence number: 40). Cytological examination of bronchial brush specimens (Figure 2D) demonstrated numerous multinucleated giant cells and histiocytes, with no evidence of malignancy. Histopathological analysis of a mediastinal mass (Figure 2E) showed abundant histiocytes, erythrocytes, and scattered inflammatory cells, including multinucleated giant cells, neutrophils, and lymphocytes. Immunohistochemical staining revealed histiocytes positive for Vimentin, CD68, and CD163, with a Ki‐67 proliferation index of 60%. Lymphocytes were LCA‐positive. Pulmonary tissue examination (Figure 2F) showed extensive necrosis with scattered atypical cells, which were positive for CXP and P40 but negative for other markers. During this period, the patient experienced persistent fever (36.5°C–38.5°C) despite ongoing antimicrobial treatment (Vancomycin 1 g Q8 h, Imipenem‐tromethamine 0.5 g Q6 h, Caspofungin 0.05 g Qd).

Days 16–32: Re‐examination of chest CT (Figure 2G) showed stable cystic pulmonary lesions. Bone marrow biopsy suggested infection with eosinophilia. Following multidisciplinary team (MDT) review, the diagnosis favored eosinophilic granulomatosis with polyangiitis (EGPA). Then treatment with methylprednisolone (80 mg Bid) and cyclophosphamide (2 mg/kg/d) was initiated, resulting in clinical improvement with defervescence and eosinophil count normalization (EO: 0.08 × 10^9^/L; EO%: 0). The patient was discharged on day 33 with oral methylprednisolone and a scheduled follow‐up. Serial hematologic parameters throughout hospitalization are detailed in Table 1.

## 3 Follow‐Up and Outcome

During the first follow‐up (Days 61), the patient required hospitalization at a local hospital for worsening respiratory symptoms, including progressive cough, purulent sputum, febrile episodes, and new hemoptysis. Laboratory tests demonstrated significant leukocytosis (WBC 66.25 × 10^9^/L) with predominant neutrophilia (92%) and concurrent eosinophilia (2.77 × 10^9^/L). Due to the discrepancy between the initial EGPA diagnosis and disease progression, a nationwide telemedicine MDT consultation was organized. According to the patient's left lower limb mass from childhood, which then ruptured and invaded the bone marrow of the left lower limb, as well as clinical manifestations and pulmonary imaging manifestations, MDT caused suspicion of non‐tuberculous mycobacterial (NTM) infection, leading to treatment modification with triple antimicrobial therapy (rifampicin 0.6 g Qd, clarithromycin 0.5 g Qd, and amikacin 0.4 g Bid) alongside continued immunosuppression (methylprednisolone 40 mg Qd, cyclophosphamide 2 mg/kg/d).

**FIGURE 3:**
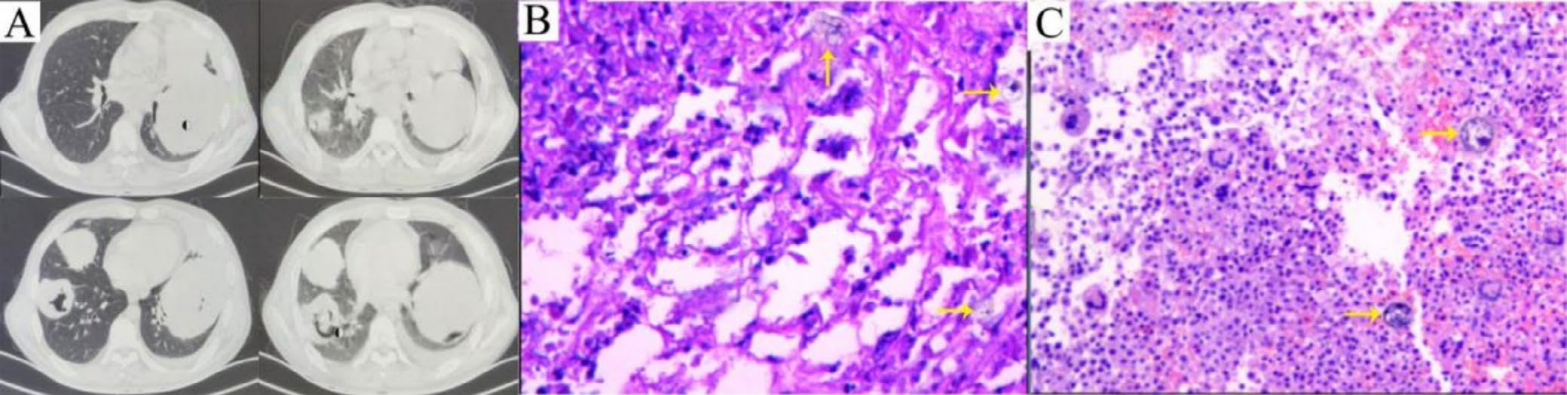
Auxiliary examinations during the follow‐up period. (A) Non‐contrast thoracic CT (Days 100). (B) Lung tissue (the yellow arrow indicates tumor cells of HS, Hematoxylin and Eosin staining, ×400) from the consultation of the the national pathology consultation institution. (C) The mass in area 7 of the trachea (the yellow arrow indicates tumor cells of HS, Hematoxylin and Eosin staining, ×400) from the consultation of the the national pathology consultation institution.

During the second follow‐up (Days 80), the patient's condition continued to deteriorate despite therapeutic interventions. A multidisciplinary review integrating the clinical course, histopathology, and literature prompted suspicion of HS. Subsequently, lung tissue specimens and bronchial mass pathology slides were referred for tertiary consultation at a national pathology consultation institution. Re‐examination of the chest CT (Figure 3A) revealed persistent multiple cystic lung lesions. The patient subsequently died of respiratory failure (Days 100). Postmortem pathological consultation from the national pathology consultation institution confirmed the HS diagnosis (Days 107, Figure 3B,C). Table 1 presents the serial peripheral blood cell count abnormalities from two follow‐up assessments.

## 4 Differential Diagnosis

EGPA is a rare type of anti‐neutrophil cytoplasmic antibody (ANCA)‐associated vasculitis [7]. The diagnostic criteria for EGPA require fulfillment of at least four out of six characteristic features: asthma, eosinophilia (> 10% in peripheral blood), peripheral neuropathy, non‐fixed pulmonary infiltrates, paranasal sinus abnormalities, and histopathological evidence of extravascular eosinophil infiltration, with a diagnostic sensitivity of 85% and specificity of 99.7% [8]. In the present case, several factors argue against the diagnosis of EGPA. First, histopathological examination revealed no evidence of vasculitis. Second, apart from pulmonary infiltrates, the patient failed to meet the remaining five diagnostic criteria. Furthermore, the lack of clinical response to EGPA‐directed therapy further supports the exclusion of this diagnosis. Similarly, while NTM infection was considered, the absence of microbiological evidence and therapeutic response precluded this diagnosis. Therefore, both EGPA and NTM infection were reasonably excluded from the differential diagnosis.

## 5 Discussion

We report this case of HS misdiagnosed as EGPA and NTM, primarily to highlight the importance of MDT and repeated histopathological assessment in rare malignant tumors, and to enhance our knowledge about this highly misleading rare malignant tumor. When clinical evolution is discordant, we change our clinical thinking in time and find the ultimate goal through clues in clinical information, thereby saving the patient's life and improving the prognosis.

The clinical diagnosis of HS is difficult, mainly relying on cytological morphology and molecular phenotypes to make a molecular pathological diagnosis. Generally, HS typically presents as a well‐demarcated or infiltrative mass, accompanied by varying degrees of hemorrhage or necrosis [9]. Histologically, it is composed of sheets of loosely arranged large polygonal cells with epithelioid pleomorphic morphology. The cytoplasm is rich, showing eosinophilic vacuolated or foamy appearance, and the nuclei are oval or irregular in shape with inconspicuous nucleoli. Occasionally, focal areas of spindle‐shaped cells can be seen. Mitotic activity and tumor necrosis are significant, and the background often shows inflammatory cell infiltration [9, 10]. Immunohistochemistry is crucial for the diagnosis of histiocytic sarcoma, which not only supports the diagnosis of HS but also differentiates it from other histiocytic‐derived malignant tumors. CD68, CD163, and lysozyme are recommended as the diagnostic cornerstones for HS [11]. Although Ki‐67, CD68, and CD163 showed high positive rates in this case, the highly indolent clinical manifestations and our limited understanding of HS led to a focus on infection. CD1a and langerin (for Langerhans cells), CD21 and CD35 (for follicular dendritic cells), CD13 and MPO (for myeloid cells), SOX10, HMB‐45, and MART‐1 (for melanocytes), keratin and EMA (for epithelium), ERG (for vessels), as well as CD20, PAX5, and CD3 (for specific B‐cells and T‐cells) markers are negative. Genetically, due to its rarity, its genetic characteristics have not been fully elucidated. Some studies have shown that there are BRAF mutations in HS, including V600 E5, 15, and non‐V600 E mutations [12], and KRAS mutations have also been reported [13].

HS is highly invasive, progresses rapidly, has a high mortality rate, and a poor prognosis, with no clear survival period. Currently, the optimal treatment strategy is unclear. For localized HS, surgery combined with chemotherapy is often used, but the treatment outcomes vary. Disseminated HS can be treated with systemic chemotherapy or a combination of chemotherapy and radiotherapy, generally choosing a chemotherapy regimen based on the CHOP protocol. Recent studies have shown that alterations in the MAP kinase pathway and chromatin regulation are fundamental to the pathogenesis of various histiocytic neoplasms (including HS), which may become future therapeutic targets [14].

Owing to its rarity and diagnostic complexity, HS exhibits a relatively high clinical misdiagnosis rate. Non‐contrast thoracic CT is mostly manifested as a large soft tissue mass in the lungs, with clear or blurred boundaries; some of the mass is lobular, the density of the mass is uneven, necrotic and liquefied areas are often seen inside, and calcification can occur in a few cases. Contrast‐enhanced chest CT is manifested as annular strengthening or patchy strengthening of uneven thickness of the tumor, and the central area is not obvious or slightly strengthening. However, in the case we report, the chest CT is mainly manifested as a cystic mass, but the strengthening is not obvious. Diagnostic challenges primarily stem from morphological similarities with other malignancies, particularly significant overlap with anaplastic large cell lymphoma (ALCL) and diffuse large B‐cell lymphoma (DLBCL) [15]. Immunophenotypic cross‐reactivity further complicates diagnosis, with approximately 18% of HS cases demonstrating aberrant expression of CD4/CD45 and other lymphoid markers [11]. The advent of molecular diagnostics, particularly next‐generation sequencing, has revolutionized the diagnostic approach to rare and frequently misdiagnosed diseases. This enables early diagnosis and treatment, which not only lowers misdiagnosis rates but also reduces mortality. Although the rapid disease progression in this case delayed definitive diagnosis, this clinical experience underscores the importance of maintaining diagnostic vigilance when clinical manifestations, treatment responses, and histopathological findings demonstrate significant discordance. Clinicians should be prepared to challenge established diagnoses, consult current literature, and pursue alternative diagnostic pathways to establish accurate diagnoses and implement targeted therapeutic strategies. This case report aims to enhance clinical awareness and diagnostic acumen for HS.

## 6 Conclusion

This patient developed a small left calf mass at 15 years of age, which gradually enlarged, ulcerated, invaded the left lower limb bone marrow, and ultimately caused amputation. During hospitalization, review of the patient's local hospital left lower limb pathological sections showed consistent morphology between calf and lung puncture lesions (inflammatory necrosis, eosinophilic abscess), initially suggesting autoimmune diseases or special infections. Combined with clinical manifestations and key findings, the primary diagnosis focused on infectious/inflammatory diseases, omitting rare malignancies like HS. During treatment, inconsistent clinical progression, poor therapeutic response, and worsening condition were noted. HS was finally confirmed via literature review, MDT discussion, and national pathological consultation center re‐evaluation. However, HS's extreme aggressiveness and rapid progression led to lost pre‐diagnosis treatment opportunities. HS typically presents at advanced stages, with limited chemotherapy response and high mortality; no standard treatment exists to date.

## Author Contributions

**Ling Zhang:** conceptualization, data curation, writing – review and editing. **Jing Qin:** conceptualization, writing – review and editing. **Peiwu Li:** formal analysis, methodology, writing – review and editing.

## Consent

A written informed consent was obtained in accordance with the journal's patient consent policy.

## Conflicts of Interest

The authors declare no conflicts of interest.

## Data Availability

The data underpinning the findings of this study are available from the corresponding author upon reasonable request. Public availability of the dataset is limited due to privacy and ethical concerns.
